# Comparison of patterns and prognosis among distant metastatic breast cancer patients by age groups: a SEER population-based analysis

**DOI:** 10.1038/s41598-017-10166-8

**Published:** 2017-08-23

**Authors:** Meng-Ting Chen, He-Fen Sun, Yang Zhao, Wen-Yan Fu, Li-Peng Yang, Shui-Ping Gao, Liang-Dong Li, Hong-lin Jiang, Wei Jin

**Affiliations:** 1 Department of Breast Surgery, Key Laboratory of Breast Cancer in Shanghai, Collaborative Innovation Center of Cancer Medicine, Fudan University Shanghai Cancer Center, Shanghai, 200030 China; 2 Department of Oncology, Shanghai Medical College, Fudan University, Shanghai, 200030 China; 3 Department of Pathology, School of Basic Medical Sciences, Fudan University, Shanghai, 200030 China; 4 Division of Molecular Medicine & Genetics, Department of Internal Medicine and Life Sciences Institute, University of Michigan, Ann Arbor, Michigan 48109 USA

## Abstract

To investigate the effects of age at diagnosis on metastatic breast cancer and patients’ prognosis, we collected patient data from the Surveillance, Epidemiology, and End Results (SEER) database. We finally identified 4932 eligible metastatic breast cancer patients diagnosed between 2010–2013, including 850 younger patients (<50 years), 2,540 middle-aged patients (50–69 years) and 1,542 elder patients (>69 years). The results revealed that in stage IV patients, elder patients were more likely to have lung metastasis (P < 0.001) and less likely to have only distant lymphatic spread (P = 0.004). Higher proportion of younger (34.9%) and middle-aged (36.2%) patients had multiple metastatic sites than elder patients (28.3%) (P < 0.001). In survival analysis, younger patients presented the best prognosis, while elder patients had the worst both in overall survival (χ^2^ = 121.9, P < 0.001) and breast cancer-specific survival (χ^2^ = 69.8, P < 0.001). Age at diagnosis was an independent prognostic factor for metastatic breast cancer patients. Moreover, patients with bone metastasis only had superior survival compared to other metastatic patients (P < 0.001). Brain metastasis only group and multiple sites metastasis group had the poorest prognosis (P < 0.05). We hope the results will provide insights into a better understanding of distant metastatic breast cancer.

## Introduction

Breast cancer is the most prevalent type of cancer among females in many countries in the past few years. It was estimated that there were 246,660 new female breast cancer patients in 2016. The overall 5-year relative survival rate is about 89% in the United States. The median age at diagnosis is 61 years, younger than many other kinds of cancer^1^. Although most breast cancer patients were diagnosed over 60 years, more and more patients were diagnosed at a younger age in the past decade^2^.

Despite the relatively high 5-year survival rate compared to other malignant tumors, distant metastasis has long been the principal cause of mortality among breast cancer patients. The most common metastatic organs were bone, lung and liver. The effective treatment involves systemic chemotherapy, endocrinotherapy and targeted therapy. However, most of the patients still have poor prognosis after metastasis. Previous study reviewed the breast cancer-specific survival (BCSS) at 10 years in primary stage IV female patients. The 10-year survival was 15.7% for ages 40 and below, 14.9% for ages 41 to 50 and 11.7% for ages 51 to 70^3^. In order to prevent and treat cancer metastasis more precisely, we have to learn more about their clinical features. The disparities of survival time among metastatic breast cancer patients were greatly associated with various clinical indicators such as pathological subtypes^4^, tumor volumes^5^, nodal status^6^, etc.

Cancer metastasis has caused enormous burden on young women patients. Some researchers found that the age of diagnosis probably played important roles in the prognosis of breast cancer^7, 8^, but few researches focused on the roles of age on metastatic patients. Knowledge of disparities in metastatic patterns may be helpful to make diagnosis of metastasis and treatment decision. In our study, we divided the metastatic patients included into three age groups, namely the younger group (<50 years), middle-aged group (50–69 years) and elder group (>69 years). Previous investigation revealed that breast cancer in younger patients may have more aggressive biological behaviours^9^. Also, elderly patients with triple-negative breast cancer (TNBC) showed higher early mortality compared to younger counterparts in the first two years of diagnosis^10^. Therefore, we aimed to identify the clinical characteristics critical and relevant to distant metastatic breast cancer by age groups in a large population via the Surveillance, Epidemiology, and End Results (SEER) database. We hope the results will contribute to the diagnosis and prevention of breast cancer progression.

## Results

### Demographics and clinical characteristics of metastatic breast cancer patients by age groups

Overall, 4932 metastatic breast cancer patients were included in our study, among which 850 (5.0%) patients were diagnosed below 50 years, 2,540 (63.7%) patients between 50 and 69 years, and 1,542 (31.2%) patients over 70 years. The median age at diagnosis was 62 years and the overall median follow-up time and interquartile range (IQR) was 10 months (2–22 months). The median observation time was 14, 11 and 6 months for the three age groups respectively. In younger group, 329 patients died at the end of the study and 296 died of breast cancer directly. The numbers were 1,200 and 1,007 respectively in middle-aged group, 914 and 727 in elder group. Table 1 summarised the frequency and proportion of some characteristics of these patient groups. There were a series of significant differences among the cohorts of patient samples including race, T stage, N stage, molecular subtypes, surgery and radiation therapy, histological types, etc. (P < 0.05). Year of diagnosis and gender had no substantive differences across the three groups. Specifically, more White people tended to have metastatic breast cancer at older age (68.0% vs. 72.6% vs. 82.2% respectively in younger, middle-aged and elder group, P < 0.05). On the contrary, Black people tended to have metastatic breast cancer at younger age (21.4% vs. 18.6% vs. 13.5% respectively, P < 0.05). Generally, younger and middle-aged patients had bigger tumor size (69.6% vs. 60.6% vs. 52.0% respectively between T2 and T4, P < 0.001) and higher rate of lymph node involvement than elder group (70.6% vs. 61.5% vs. 47.8% respectively, P < 0.001). The T stage and N stage of a few patients were unknown from SEER database. Therefore, the results may need further validation. Moreover, breast cancer molecular subtypes were also important indicators for treatment and prognosis. These three age groups also showed different molecular subtype patterns. Triple negative breast cancer (TNBC) is usually more aggressive than other subtypes and there were limited therapies for TNBC so far. It was noticed that younger patients had obviously higher rate of TNBC (13.1% vs. 10.9% vs. 9.3% respectively, P < 0.05) in our study. As for treatment, younger and middle-aged patients had significantly higher rate of surgery and radiation treatment compared to elder counterparts (P < 0.001). The results may be attributed to their better physical condition to withstand the treatment. In addition, the histological types also showed differences in stage IV patients by age groups (P < 0.001). The most common histological type was infiltrating duct carcinoma (IDC) (younger vs. middle-aged vs. elder: 65.8% vs. 55.0% vs. 46.0% respectively). The second and third common histological types were carcinoma *in situ* (7.2%) and adenocarcinoma (6.9%) in younger group, adenocarcinoma (12.1%) and lobular carcinoma (10.8%) in middle-aged group, adenocarcinoma (15.2%) and carcinoma *in situ* (14.0%) in elder group.

**Table 1:** Characteristics of breast cancer patients with distant metastasis from SEER 18 population-based registries by age groups.

		Age < 50 years n = 850 (100%)		50–69 years n = 2,540 (100%)		Age > 69 years n = 1,542 (100%)		Total n = 4,932(100%)		P-value^a^
		No.	%	No.	%	No.	%	No.	%	
Gender	Female	841	98.9%	2,512	98.9%	1,519	98.5%	4,872	98.8%	0.491
	Male	9	1.1%	28	1.1%	23	1.5%	60	1.2%	
Year of diagnosis	2010	205	24.1%	639	25.2%	357	23.2%	1,201	24.4%	0.432
	2011	225	26.5%	624	24.6%	380	24.6%	1,229	24.9%	
	2012	221	26.0%	634	25.0%	387	25.1%	1,242	25.2%	
	2013	199	23.4%	643	25.3%	418	27.1%	1,260	25.5%	
Race	White	578	68.0%	1,843	72.6%	1,267	82.2%	3,688	74.8%	**<0.001**
	Black	182	21.4%	472	18.6%	208	13.5%	862	17.5%	
	Others^b^	87	10.2%	207	8.1%	59	3.8%	353	7.2%	
	Unknown	3	0.4%	18	0.7%	8	0.5%	29	0.6%	
T stage	T0	23	2.7%	144	5.7%	109	7.1%	276	5.6%	**<0.001**
	T1	63	7.4%	168	6.6%	92	6.0%	323	6.5%	
	T2	157	18.5%	289	11.4%	189	12.3%	635	12.9%	
	T3	108	12.7%	249	9.8%	129	8.4%	486	9.9%	
	T4	326	38.4%	1,001	39.4%	483	31.3%	1,810	36.7%	
	Tx	173	20.4%	689	27.1%	540	35.0%	1,402	28.4%	
N stage	N0	149	17.5%	534	21.0%	418	27.1%	1,101	22.3%	**<0.001**
	N1	357	42.0%	910	35.8%	481	31.2%	1,748	35.4%	
	N2	91	10.7%	271	10.7%	119	7.7%	481	9.8%	
	N3	152	17.9%	380	15.0%	137	8.9%	669	13.6%	
	Nx	101	11.9%	445	17.5%	387	25.1%	933	18.9%	
Molecular subtype^c^	Her2−/HoR+	402	47.3%	1,228	48.4%	737	47.8%	2,367	48.0%	**<0.001**
	Her2 + /HoR +	148	17.4%	308	12.1%	130	8.4%	586	11.9%	
	Her2+/HoR−	82	9.6%	185	7.3%	72	4.7%	339	6.9%	
	Triple Negative	111	13.1%	277	10.9%	143	9.3%	531	10.8%	
	Unknown	107	12.6%	541	21.3%	460	29.8%	1,109	22.5%	
Surgery^d^	No	542	63.8%	1,910	75.2%	1,273	82.6%	3,725	75.5%	**<0.001**
	Yes	301	35.4%	611	12.4%	259	16.8%	1,171	23.7%	
	Unknown	7	0.8%	19	0.7%	10	0.6%	36	0.7%	
Radiation	No	517	60.8%	1,743	68.6%	1,161	75.3%	3,421	69.4%	**<0.001**
	Yes	305	35.9%	728	28.7%	342	22.2%	1,375	27.9%	
	Unknown	28	3.3%	69	2.7%	39	2.5%	136	2.8%	
Median follow-up (months)		14 (5–26)		11 (3–22)		6 (1–18)		10 (2–22)		

^a^The bold type indicates statistically significance.

^b^Other races includes American Indian, AK Native, Asian and Pacific Islander.

^c^Her2: human epidermal growth factor receptor-2; HoR: hormone receptor.

^d^The surgery only included surgery at the primary site.

### Different patterns of metastasis in breast cancer patients by age groups

Among the study population, we found that bone was still the most common site of metastasis for breast cancer (65.1%, including single and multiple metastatic sites), followed by lung (31.4%), liver (26.0%) and brain (8.8%) metastasis. Patients with multiple organs metastasis usually had fewer treatment options and tended to had poorer outcomes. Unfortunately, at least 33.5% of all the cases had multiple organs metastasis. The most common multiple metastatic combination was bone and lung, constituting 31.4% (n = 484) of the multiple metastatic patients.

We then identified 2,881 single site and 1,653 multiple organs metastatic patients. (Table 2). Interestingly, among patients with single metastatic site, elder patients had significantly higher rate of lung metastasis (5.9% vs. 7.6% vs. 14.2% respectively, P < 0.001) and lower rate of only distant lymphatic metastasis (7.3% vs. 5.4% vs. 4.0% respectively, P = 0.004). We also found that higher proportion of younger (34.9%) and middle-aged (36.2%) patients had multiple metastatic sites than elder patients (28.3%) (P < 0.001). However, there were no significant differences in bone, liver or brain only metastasis among the three age groups.

**Table 2:** The number and proportion of breast cancer patients with single metastatic site and multiple metastatic sites.

	Age < 50 years		50–69 years		Age > 69 years		Total		P-value
	No.	%	No.	%	No.	%	No.	%	
Bone only	307	36.1%	868	34.2%	581	37.7%	1,756	35.6%	0.072
Lung only	50	5.9%	192	7.6%	219	14.2%	461	9.3%	**<0.001**
Liver only	69	8.1%	160	6.3%	88	5.7%	317	6.4%	0.066
Brain only	16	1.9%	46	1.8%	24	1.6%	86	1.7%	0.787
Distant lymph nodes only	62	7.3%	136	5.4%	63	4.0%	261	5.3%	**0.004**
Multiple sites	297	34.9%	920	36.2%	436	28.3%	1,653	33.5%	**<0.001**

### Comparison of overall survival (OS) and breast cancer-specific survival (BCSS) among the study population

**Figure 1:**
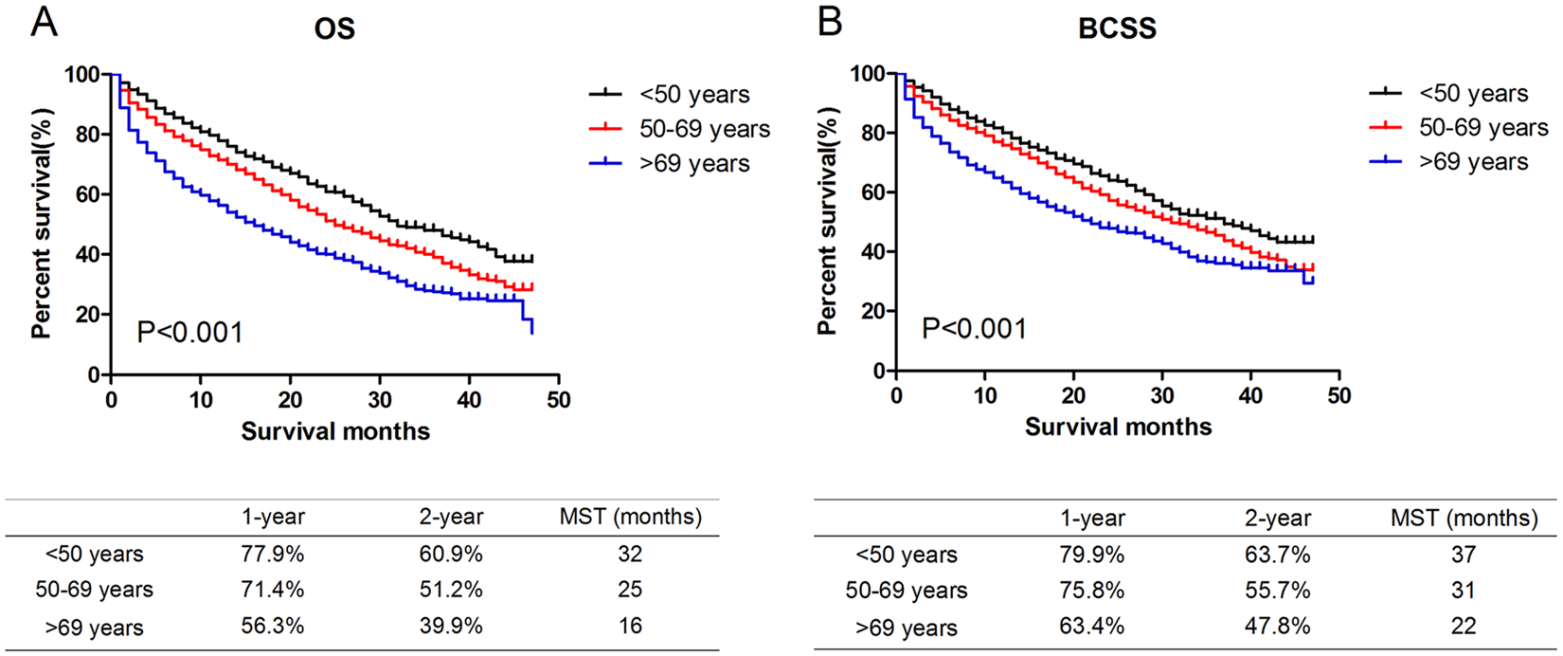
Comparison of survival in younger, middle-aged and elder metastatic breast cancer patients. Kaplan Meier analysis for overall survival (OS, χ^2^ = 121.9, P < 0.001, Fig. 1A) and breast cancer-specific survival (BCSS, χ^2^ = 69.8, P < 0.001, Fig. 1B) were shown in the graph. The prognosis became worse with the increase of age. The 1-year, 2-year survival rate and median survival time (MST) were listed respectively in the table below the graph.

As shown in Kaplan-Meier plots (Fig. 1), there were substantive differences in OS (χ^2^ = 121.9, P < 0.001) and BCSS (χ^2^ = 69.8, P < 0.001) among the three age groups. Younger patients presented the best outcome while elder patients had the worst. The median overall survival time was 32, 25 and 16 months respectively in younger, middle-aged and elder groups.

Moreover, we conducted univariate analysis and multivariate analysis (Table 3) with Cox hazard regression model to evaluate the effects of baseline characteristics on OS and BCSS in the whole study population. In the multivariate analysis, we found that age at diagnosis, race, T stage, molecular subtypes, surgery, radiation therapy, and distant organ metastasis were all significantly associated with BCSS (P < 0.05). All of the factors above were associated with OS (P < 0.05) except bone metastasis (P = 0.299). However, gender, year of diagnosis and N stage were not distinctly correlated with prognosis in this model. As was shown, age at diagnosis was an independent prognostic factor for metastatic breast cancer patients. Compared to middle-aged patients, younger patients had better OS (HR: 0.77, 95% CI: 0.68–0.87, P < 0.001) and BCSS (HR: 0.81, 95% CI: 0.71–0.92, P = 0.002). The elder group had the worst OS (HR: 1.56, 95% CI: 1.43–1.70, P < 0.001) and BCSS (HR:1.52, 95% CI: 1.38–1.68, P < 0.001). The results were consistent with Kaplan-Meier plots. We also found that patients underwent primary site surgery or radiotherapy had better survival, indicating potential benefits from regional treatment in metastatic patients.

**Table 3:** Univariate and multivariate analysis of overall survival (OS) and breast cancer-specific survival (BCSS) of the study population.

		Univariate analysis				Multivariate analysis			
		OS		BCSS		OS		BCSS	
		HRs (95% CI)^a^	P-value	HRs (95% CI)	P-value	HRs (95% CI)	P-value	HRs (95% CI)	P-value
Age	**<**50 years	0.72 (0.64–0.82)	**P < 0.001**	0.78 (0.68–0.88)	**P < 0.001**	0.77 (0.68–0.87)	**P < 0.001**	0.81 (0.71–0.92)	**0.002**
	50–69 years	reference	—	reference	—	reference	—	reference	—
	>69 years	1.50 (1.38–1.64)	**P < 0.001**	1.43 (1.30–1.57)	**P < 0.001**	1.56 (1.43–1.70)	**P < 0.001**	1.52 (1.38–1.68)	**P < 0.001**
Gender	Female	reference	—	reference	—	reference	—	reference	—
	Male	1.04 (0.71–1.51)	0.850	1.03 (0.68–1.55)	0.907	0.96 (0.66–1.40)	0.962	0.99 (0.65–1.50)	0.954
Year of diagnosis	2010	reference	—	reference	—	reference	—	reference	—
	2011	0.93 (0.84–1.03)	0.175	0.91 (0.82–1.02)	0.913	0.94 (0.85–1.04)	0.214	0.91 (0.82–1.02)	0.111
	2012	0.97 (0.86–1.08)	0.532	0.96 (0.85–1.02)	0.481	0.98 (0.88–1.10)	0.714	0.97 (0.86–1.10)	0.605
	2013	0.94 (0.82–1.08)	0.402	0.94 (0.81–1.10)	0.440	0.92 (0.80–1.06)	0.230	0.91 (0.78–1.06)	0.241
Race	White	reference	—	reference	—	reference	—	reference	—
	Black	1.20 (1.09–1.33)	**P < 0.001**	1.17 (1.05–1.31)	**0.006**	1.22 (1.10–1.36)	**P < 0.001**	1.15 (1.03–1.29)	**0.015**
	Others	0.83 (0.70–0.98)	0.023	0.87 (0.73–1.04)	0.116	0.91 (0.77–1.08)	0.270	0.93 (0.78–1.11)	0.419
	Unknown	0.35 (0.13–0.95)	**0.038**	0.43 (0.16–1.13)	0.088	0.44 (0.17–1.19)	0.443	0.56 (0.21–1.49)	0.244
T stage	T0	reference	—	reference	—	reference	—	reference	—
	T1	0.67 (0.52–0.87)	**0.003**	0.79 (0.58–1.07)	0.121	0.99 (0.76–1.29)	0.934	1.13 (0.83–1.54)	0.426
	T2	0.85 (0.69–1.06)	0.146	1.07 (0.83–1.39)	**0.588**	1.27 (1.02–1.59)	0.035	1.55 (1.19–2.01)	**0.001**
	T3	0.94 (0.75–1.18)	0.617	1.28 (0.99–1.67)	0.061	1.34 (1.06–1.70)	**0.014**	1.77 (1.35–2.31)	**P < 0.001**
	T4	1.28 (1.06–1.55)	**0.011**	1.66 (1.32–2.09)	**P < 0.001**	1.62 (1.33–1.98)	**P < 0.001**	2.02 (1.60–2.57)	**P < 0.001**
	Tx	1.44 (1.19–1.75)	**P < 0.001**	1.69 (1.34–2.14)	**P < 0.001**	1.44 (1.18–1.75)	**P < 0.001**	1.68 (1.32–2.13)	**P < 0.001**
N stage	N0	reference	—	reference	—	reference	—	reference	—
	N1	0.93 (0.84-1.04)	0.208	1.03 (0.92–1.17)	0.592	0.91 (0.81–1.02)	0.088	0.96 (0.85–1.09)	0.503
	N2	0.78 (0.66–0.91)	**0.001**	0.85 (0.71–1.01)	0.060	0.87 (0.73–1.03)	0.095	0.90 (0.75–1.08)	0.241
	N3	0.92 (0.80–1.06)	0.241	1.02 (0.88–1.19)	0.757	1.00 (0.87-1.16)	0.975	1.05 (0.90–1.24)	0.5336
	Nx	1.34 (1.19–1.50)	**P < 0.001**	1.35 (1.18–1.54)	**P < 0.001**	1.07 (0.95–1.21)	0.285	1.08 (0.94–1.24)	0.293
Molecular subtype	Her2–/HoR +	reference	—	reference	—	reference	—	reference	—
	Her2 + /HoR+	0.88 (0.76-1.02)	0.085	0.86 (0.73–1.01)	0.061	0.86 (0.74–1.00)	**0.035**	0.81 (0.69–0.95)	**0.011**
	Her2+/HoR–	1.17 (0.99–1.38)	0.073	1.18 (0.99–1.42)	0.066	1.08 (0.91–1.28)	0.368	1.08 (0.89–1.29)	0.439
	Triple Negative	2.29 (2.03–2.58)	**P < 0.001**	2.33 (2.05-0.65)	**P < 0.001**	2.26 (2.00–2.56)	**P < 0.001**	2.31 (2.02–2.64)	**P < 0.001**
	Unknown	1.78 (1.61–1.96)	**P < 0.001**	1.64 (1.47–1.83)	**P < 0.001**	1.53 (1.38–1.70)	**P < 0.001**	1.48 (1.32–1.66)	**P < 0.001**
Surgery	No	reference	—	reference	—	reference	—	reference	—
	Yes	0.49 (0.44-0.55)	**P < 0.001**	0.51 (0.45–0.57)	**P < 0.001**	0.61 (0.55–0.69)	**P < 0.001**	0.62 (0.55–0.70)	**P < 0.001**
	Unknown	1.21 (0.77–1.90)	0.414	1.31 (0.81-2.11)	0.270	1.54 (0.96–2.45)	0.071	1.64 (1.00–2.69)	0.051
Radiation	No	reference	—	reference	—	reference	—	reference	—
	Yes	0.72 (0.66–0.79)	**P < 0.001**	0.75 (0.68–0.83)	**P < 0.001**	0.79 (0.72–0.87)	**P < 0.001**	0.80 (0.72–0.89)	**P < 0.001**
	Unknown	0.65 (0.49-0.86)	**0.003**	0.67 (0.49–0.91)	**0.010**	0.77 (0.58–1.03)	0.080	0.77 (0.56–1.06)	0.109
Bone metastasis	No	reference	—	reference	—	reference	—	reference	—
	Yes	0.92 (0.85-1.00)	**0.040**	0.97 (0.87–1.06)	0.970	1.05 (0.96–1.15)	0.267	1.11 (1.01–1.22)	**0.035**
Lung metastasis	No	reference	—	reference	—	reference	—	reference	—
	Yes	1.47 (1.36-1.60)	**P < 0.001**	1.52 (1.39–1.67)	**P < 0.001**	1.22 (1.12–1.33)	**P < 0.001**	1.24 (1.13-1.36)	**P < 0.001**
Liver metastasis	No	reference	—	reference	—	reference	—	reference	—
	Yes	1.79 (1.64–1.94)	**P < 0.001**	1.90 (1.73–2.09)	**P < 0.001**	1.80 (1.65-1.97)	**P < 0.001**	1.90 (1.72–2.09)	**P < 0.001**
Brain metastasis	No	reference	—	reference	—	reference	—	reference	—
	Yes	1.81 (1.60–2.05)	**P < 0.001**	1.87 (1.64-2.14)	**P < 0.001**	1.74 (1.53–1.98)	**P < 0.001**	1.77 (1.54–2.04)	**P < 0.001**

^a^HRs: hazard ratios; CI: confidence interval.

**Figure 2:**
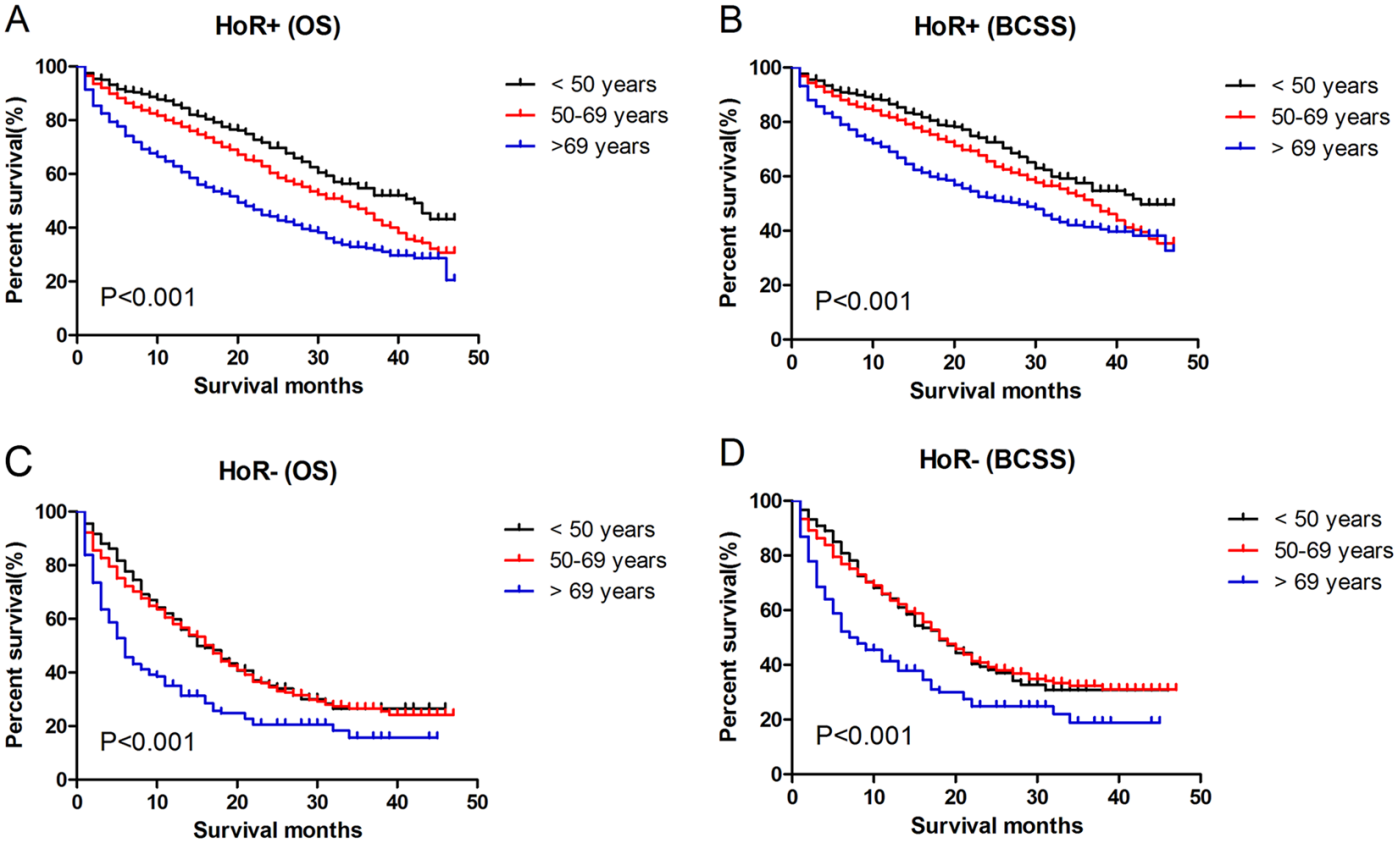
Comparison of survival in breast cancer patients with different hormone receptor status. Kaplan Meier analysis for OS and BCSS in different subgroups were shown in the graph. In hormone receptor positive (**A**,**B**) patients, the prognosis became worse with the increase of age (P < 0.001). In hormone receptor negative patients (**C**,**D**), middle-aged patients had similar survival to younger patients (P > 0.05), though older patients still had the worst prognosis. HoR+: hormone receptor positive; HoR-: hormone receptor negative.

We also conducted survival analysis in subgroups according to hormone receptor status (Fig. 2). In hormone receptor positive patients, the prognosis became worse with the increase of age. However, in hormone receptor negative patients, younger group did not show better prognosis compared to middle-aged patients (P > 0.05). The middle-aged group even had slightly longer median survival time (MST) than younger group.

### Comparison of survival among single site and multiple sites metastatic breast cancer patients

**Figure 3:**
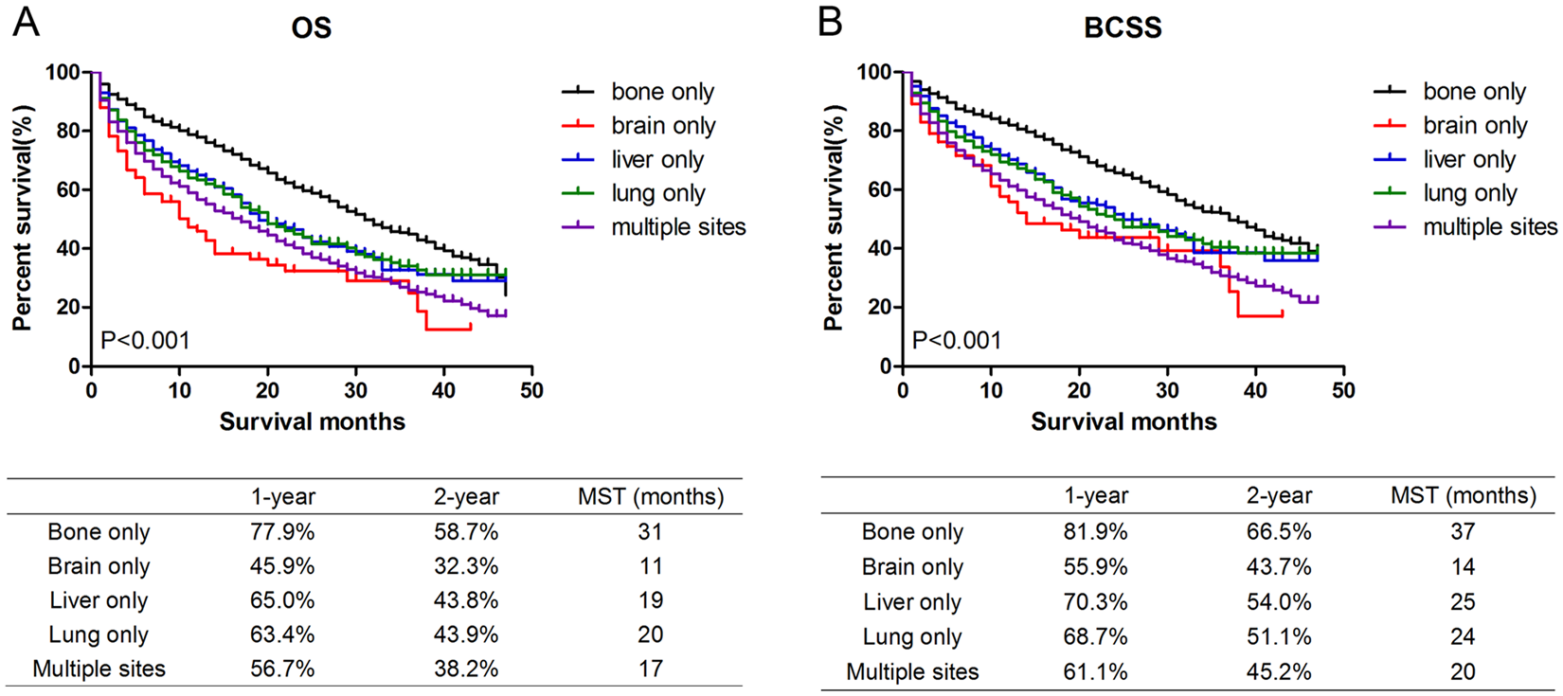
Comparison of survival in breast cancer patients with single or multiple metastatic sites. Kaplan Meier analysis for OS (χ^2^ = 147.7, P < 0.001, Fig. 2A) and BCSS (χ^2^ = 145.7, P < 0.001, Fig. 2B) were shown. The 1-year, 2-year survival rate and median survival time (MST) were listed respectively in the table below the graph. MST: median survival time.

We used Kaplan-Meier analysis to compare the effects of single and multiple distant metastatic organs on survival time among the study population. The 1-year and 2-year survival rate and MST were also calculated for each group. The results suggested that there were significant differences among patients with different specific metastatic sites in OS (χ^2^ = 147.7, P < 0.001, Fig. 3A) and BCSS (χ^2^ = 145.7, P < 0.001, Fig. 3B). Patients with bone metastasis only had superior survival compared to other metastatic patients (MST: 31 months in OS and 37 months in BCSS, P < 0.001). Patients with lung or liver invasion only had similar intermediate MST (P > 0.05). However, brain metastasis only group (MST: 11 months in OS and 14 months in BCSS) and multiple sites metastasis group (MST: 17 months in OS and 20 months in BCSS) had the poorest prognosis compared to other groups (P < 0.05) and there was no significant difference between these two groups both in OS and BCSS (P > 0.05) as well. The prognosis of three age groups with single metastasis was also analysed (Supplementary Figure S1). The results suggested that the prognosis become worse with the increase of age in bone metastasis only and liver only patients (P < 0.001), but not in lung metastatic patients (P > 0.05). The statistics of brain metastasis was not analysed because of the limited number of samples.

## Discussion

With the increase of cancer incidence worldwide, there will be more patients suffer from cancer invasion and metastasis. Tumor metastasis is a complicated and multi-stage process which involves cell proliferation, angiogenesis, migration and many other cell functions^11^. There are certain specificities in tumor dissemination and invasion to distant organs. We hope to provide deep insights into better understanding of the heterogeneity of breast cancer.

In our retrospective study, we analysed the influence of age at diagnosis on breast cancer metastasis and mortality. The incidence of breast cancer among younger women has been increased in recent years^2^. This phenomenon may be due to the deteriorating pollution, increased exposure to estrogen, alcohol or many other risk factors. There were also significant differences between the three age groups including race, T stage, N stage, molecular subtypes, surgery and radiation therapy, histological types, etc. Interestingly, we found that younger age at diagnosis was associated with bigger tumor size, more lymph node involvement and higher rate of TNBC. Also, younger stage IV patients were more likely to have multiple and distant lymph nodes metastasis, but less likely to have lung metastasis. Our results are generally consistent with some of the previous studies^12, 13^. However, another study among 3553 breast cancer patients, with 6.32 years median follow-up, revealed a reduction in risk of bone and viscera metastasis with the increase of age at diagnosis^14^. The MST was 1.52 (IQR: 0.7–2.9) years for patients with bone metastasis and 0.7 (IQR: 1.2–1.5) years for visceral metastasis. Hung MH, *et al*. suggested that younger patients (age < 35 years) were particularly at risk of brain metastasis regardless of biological subtype^15^. The “seed and soil hypothesis” may partially account for the phenomenon of different metastatic patterns^16^. It seems that different subpopulations of tumor cells favored different microenvironment of distant organs, which provides ideal condition for their invasion and proliferation. It is necessary to treat different subpopulations of tumor cells with different strategies. The underlying molecular mechanisms still need further investigations. We also hypothesize that the immune system may have important influence on the various patterns of metastasis. The younger patients usually have stronger immune response, leading to differences in tumor microenvironment. Recent research found that neutrophils can help the colonization of breast cancer initiating cells in the lung^17^. The roles of immune cells and inflammatory mediators, like a two-edged sword, in tumor metastasis are still controversial.

We also evaluated the effects of age at diagnosis and metastatic sites on breast cancer mortality in a large sample of population. In Kaplan Meier analysis and Cox hazard regression model, the results indicated that older age contributed significantly to the poorer prognosis both in OS and BCSS. Age at diagnosis was one of independent prognostic factors in the study population. There may be multi-factorial explanations. A lot of age-related factors may play important roles in metastasis, including accumulation of DNA damage, immune response^18^, chronic inflammation^19^, hormone level changes, etc. Furthermore, younger patients usually have higher chance to receive chemotherapy, radiation therapy and surgery. Even with distant organ metastasis, patients will still benefit from local treatment, including mastectomy^20, 21^. Interestingly, in hormone receptor positive patients, younger group showed better prognosis than middle-aged patients, while the two age groups had similar survival time in hormone receptor negative patients. Recent studies revealed the important roles of hormone level on distant metastasis^22^. The disparity in hormone level may influence the incidence and survival between prepremenopausal and postmenopausal patients. Many other researches suggested that younger breast cancer patients were characterised as more advanced stages and more aggressive in clinical behaviour^23, 24^. There may be other underlying factors. All of these factors contribute to the complex relationship between age and metastasis.

More importantly, we demonstrated that patients with different metastasis patterns had different survival outcomes. To be specific, bone metastasis only group had the longest MST compared to other metastatic patients, while brain metastasis only group and multiple sites metastasis group had the poorest outcome. Patients with only lung and only liver invasion had similar MST. Despite large number of bone metastatic patients, there are several effective chemotherapies, endocrine therapy and other medications such as zoledronic acid, which have distinct benefits on stage IV patients survival. Unfortunately, effective therapies are still limited for patients with brain (mainly because of the blood-brain barrier) and multiple sites metastasis currently^25^. The results may remind clinical physicians of treating breast cancer patients in a more individual manner.

Tremendous efforts had been made to explore the prevention and treatment of breast cancer metastasis. The concept of precise medicine had been put forward recently in order to prevent and treat cancer more individually. It was recently revealed that there were circulating tumor cells (CTCs) in the peripheral blood of breast cancer patients^26^. CTCs were also estimated to be an independent prognostic indicator of poor survival outcomes for triple-negative breast cancer (TNBC) patients^27^. Breast cancer was considered to be a systematic disease at the very early stage. If the CTCs can be detected more sensitively in the blood or by other non-invasive means, distant metastasis may be predicted more precisely before lesions in distant organs appeared. Therefore, it was necessary to prevent distant metastasis at an earlier period of the disease than we thought before.

There are also some limitations in our study. We could not collect the patients’ information of chemotherapy, endocrine therapy and many other related factors from SEER database. This may cause a certain bias in our results. In addition, the information of specific metastatic organ and Her2 status was only available after 2010 in SEER database, which may not ensure enough number of samples and enough time of follow-up in the study, especially brain metastatic samples. Besides age at diagnosis, several other factors may also influence the survival time. For example, younger metastatic patients may have higher chances to achieve chemotherapy, targeted therapy or other intensive systemic treatment. Many elder patients had co-morbidities which may lead to the exacerbation of the disease. Therefore, the results need further validation in future studies.

In conclusion, our research summarised the clinical characteristics and survival outcomes of metastatic breast cancer patients in three age groups in a large sample of population. The results may provide more evidence for precise medicine and individualized therapy. Further efforts still need to be done in order to investigate more comprehensive factors associated with breast cancer metastasis in the future.

## Methods

### Ethics statement

The SEER research data files were downloaded using the reference number 11443-Nov2015. The data released by the SEER database do not require informed patient consent. Our study had already been approved by the Ethical Committee and Institutional Review Board of Fudan University Shanghai Cancer Centre (FDUSCC). The methods were performed in accordance with the approved guidelines.

### Data collection

SEER*Stat version 8.3.2 was utilized to filtrate and collect the information of representative patients in the research (http://seer.cancer.gov/). We chose patients from SEER 18 Regs Research Data which covered approximately 28% of the U.S. population when follow-up ended before 31/12/2013. We finally focused on 4932 eligible patients based on the following criteria: microscopically confirmed primary breast cancer patients, diagnosis between 2010 and 2013, known age at diagnosis, and *de novo* stage IV (AJCC 7^th^ edition) patients. Patients with metastasis to distant lymph nodes were also included. We chose patients who were diagnosed since 2010 because the information of distant metastatic to specific organs and molecular subtypes were only available after 2010. The patients registered after 2013 were not included because we would like to ensure enough time of follow-up.

### Statistical analysis

All the patients were divided into three age groups: the younger group (<50 years), middle-aged group (50–69 years) and elder group (>69 years). We used SPSS 22.0 software to analyse the information we obtained from the database. The clinical characteristics of the selected patients were compared with the Pearson’s χ2 test. The survival curves were drawn with Kaplan Meier analysis and the curves were compared with log rank test with GraphPad Prism 5.0. Cox regression models were used to identify factors which were significantly associated with overall survival (OS) and breast cancer-specific survival (BCSS). OS was defined as the time from breast cancer diagnosis to death due to any cause and BCSS from breast cancer diagnosis to death due to breast cancer. The 1-year and 2-year survival rate and median survival rate was also calculated. At the meantime, hazard ratios (HRs) and 95% confidence interval (95% CI) were also analysed. We defined P-value < 0.05 as statistically significant.

## Electronic supplementary material

Dataset 1

## References

[1] Miller KD (2016). Cancer treatment and survivorship statistics, 2016. CA: a cancer journal for clinicians..

[2] DeSantis CE (2016). Breast cancer statistics, 2015: Convergence of incidence rates between black and white women. CA: a cancer journal for clinicians..

[3] Eng LG (2016). Ten-year survival in women with primary stage IV breast cancer. Breast cancer research and treatment..

[4] Kennecke H (2010). Metastatic behavior of breast cancer subtypes. Journal of clinical oncology: official journal of the American Society of Clinical Oncology..

[5] Marinelli B (2016). Prognostic value of FDG PET/CT-based metabolic tumor volumes in metastatic triple negative breast cancer patients. American journal of nuclear medicine and molecular imaging..

[6] Wang XX, Jiang YZ, Li JJ, Song CG, Shao ZM (2016). Effect of nodal status on clinical outcomes of triple-negative breast cancer: a population-based study using the SEER 18 database. Oncotarget..

[7] Crivellari D (2007). Breast cancer in the elderly. Journal of clinical oncology..

[8] Sabiani L (2016). Breast cancer in young women: Pathologic features and molecular phenotype. Breast (Edinburgh, Scotland)..

[9] Azim HA (2012). Elucidating prognosis and biology of breast cancer arising in young women using gene expression profiling. Clinical cancer research..

[10] Zhu W, Perez EA, Hong R, Li Q, Xu B (2015). Age-Related Disparity in Immediate Prognosis of Patients with Triple-Negative Breast Cancer: A Population-Based Study from SEER Cancer Registries. PloS one..

[11] Hanahan D, Weinberg RA (2011). Hallmarks of cancer: the next generation. Cell..

[12] Inal A (2014). Pathologic and clinical characteristics of elderly patients with breast cancer: a retrospective analysis of a multicenter study (Anatolian Society of Medical Oncology). International surgery..

[13] Liedtke C (2015). The prognostic impact of age in different molecular subtypes of breast cancer. Breast cancer research and treatment..

[14] Purushotham A (2014). Age at diagnosis and distant metastasis in breast cancer–a surprising inverse relationship. European journal of cancer..

[15] Hung M. H. et al. Effect of age and biological subtype on the risk and timing of brain metastasis inbreast cancer patients. *PLoS One*. **9**, doi:10.1371/journal.pone.0089389 (2014). 10.1371/journal.pone.0089389 PMC3933537 24586742

[16] Ribelles N, Santonja A, Pajares B, Llacer C, Alba E (2014). The seed and soil hypothesis revisited: current state of knowledge of inherited genes on prognosis in breast cancer. Cancer treatment reviews..

[17] Wculek SK, Malanchi I (2015). Neutrophils support lung colonization of metastasis-initiating breast cancer cells. Nature..

[18] Yang J, Li X, Liu X, Liu Y (2015). The role of tumor-associated macrophages in breast carcinoma invasion and metastasis. International journal of clinical and experimental pathology..

[19] Hugo HJ, Saunders C, Ramsay RG, Thompson EW (2015). New Insights on COX-2 in Chronic Inflammation Driving Breast Cancer Growth and Metastasis. Journal of mammary gland biology and neoplasia..

[20] Warschkow R (2016). Improved Survival After Primary Tumor Surgery in Metastatic Breast Cancer: A Propensity-adjusted, Population-based SEER Trend Analysis. Annals of surgery..

[21] Sofi AA, Mohamed I, Koumaya M, Kamaluddin Z (2013). Local therapy in metastatic breast cancer is associated with improved survival. American journal of therapeutics..

[22] Hosseini H (2016). Early dissemination seeds metastasis in breast cancer. Nature..

[23] Kataoka A (2014). Clinicopathological features of young patients (<35 years of age) with breast cancer in a Japanese Breast Cancer Society supported study. Breast Cancer..

[24] Park YH (2015). Prevalence and clinical outcomes of young breast cancer (YBC) patients according to intrinsic breast cancer subtypes: Single institutional experience in Korea. Breast..

[25] Rostami R, Mittal S, Rostami P, Tavassoli F, Jabbari B (2016). Brain metastasis in breast cancer: a comprehensive literature review. Journal of neuro-oncology..

[26] Bidard FC, Proudhon C, Pierga JY (2016). Circulating tumor cells in breast cancer. Molecular oncology..

[27] Lu YJ (2016). The significant prognostic value of circulating tumor cells in triple-negative breast cancer: a meta-analysis. Oncotarget..

