# Supplementary material for: Comparison of patterns and prognosis among distant metastatic breast cancer patients by age groups: a SEER population-based analysis
Source: Sci Rep. 2017 Aug 23;7:9254. doi: 10.1038/s41598-017-10166-8 (PMC5569011; doi:10.1038/s41598-017-10166-8)
Supplement: Supplementary file 1 — Dataset 1 [file 41598_2017_10166_MOESM1_ESM.zip › Supplementary.pdf]

SUPPLEMENTARY IMFORMATION

Comparison of patterns and prognosis among distant metastatic breast cancer patients by age groups: a SEER population-based analysis

Meng-Ting Chen[#1,2], **He-Fen Sun**[#1,2], **Yang Zhao**[1,2], **Wen-Yan Fu**[1,2], **Li-Peng Yang**[3], **Shui-Ping Gao**[1,2], **Liang-Dong Li**[1,2], **Hong-lin Jiang**[4], **Wei Jin**[1,2]*

1. Department of Breast Surgery, Key Laboratory of Breast Cancer in Shanghai, Collaborative Innovation Center of Cancer Medicine, Fudan University Shanghai Cancer Center, Shanghai, 200030, China
2. Department of Oncology, Shanghai Medical College, Fudan University, Shanghai, 200030, China
3. Department of pathology, School of Basic Medical Sciences, Fudan University, Shanghai, 200030, China
4. Division of Molecular Medicine & Genetic, Department of internal Medicine and Life Sciences Institute, University of Michigan, Ann Abor, Michigan 48109, USA.

[#] These authors have contributed equally to this work.

*** Correspondence to：**

Wei Jin, Department of Breast Surgery, Key Laboratory of Breast Cancer in Shanghai, Fudan University Shanghai Cancer Center, Shanghai, 200030, China,

Tel: +86-21-64175590-3423; Fax: +86-21-64031696, E-mail: jinwei7207@163.com

The document includes 1 supplementary figure.

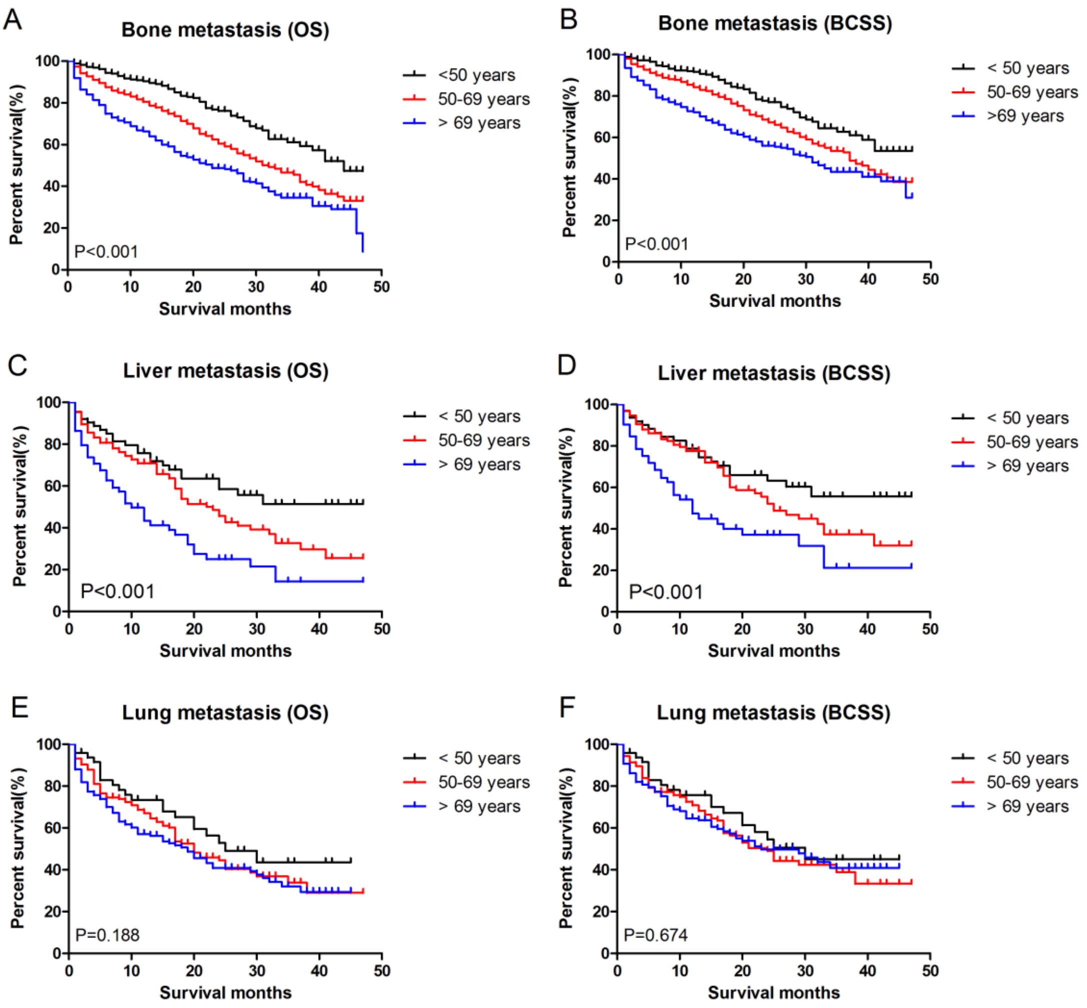

Supplementary Figure S1. Comparison of survival in breast cancer patients with single site metastasis

Kaplan Meier analysis for OS and BCSS indicated that the prognosis become worse with the increase of age in bone (A, B) and liver (C, D) metastatic patients ($P < 0.001$), but not in lung metastatic patients (E, F, $P > 0.05$). The statistics of brain metastasis was not analysed because of the limited number of samples.
